# Inhibitory activity spectrum of reuterin produced by *Lactobacillus reuteri *against intestinal bacteria

**DOI:** 10.1186/1471-2180-7-101

**Published:** 2007-11-12

**Authors:** Valentine Cleusix, Christophe Lacroix, Sabine Vollenweider, Marc Duboux, Gwenaelle Le Blay

**Affiliations:** 1 Institute of Food Science and Nutrition, Laboratory of Food Biotechnology, Swiss Federal Institute of Technology, Zurich, Switzerland

## Abstract

**Background:**

Reuterin produced from glycerol by *Lactobacillus reuteri*, a normal inhabitant of the human intestine, is a broad-spectrum antimicrobial agent. It has been postulated that reuterin could play a role in the probiotic effects of *Lb. reuteri*. Reuterin is active toward enteropathogens, yeasts, fungi, protozoa and viruses, but its effect on commensal intestinal bacteria is unknown. Moreover reuterin's mode of action has not yet been elucidated. Glutathione, a powerful antioxidant, which also plays a key role in detoxifying reactive aldehydes, protects certain bacteria from oxidative stress, and could also be implicated in resistance to reuterin.

The aim of this work was to test the activity of reuterin against a representative panel of intestinal bacteria and to study a possible correlation between intracellular low molecular weight thiols (LMW-SH) such as glutathione, hydrogen peroxide and/or reuterin sensitivity. Reuterin was produced by *Lb*. *reuteri *SD2112 in pure glycerol solution, purified and used to test the minimal inhibitory (MIC) and minimal bactericidal concentrations (MBC). Hydrogen peroxide sensitivity and intracellular LMW-SH concentration were also analysed.

**Results:**

Our data showed that most tested intestinal bacteria showed MIC below that for a sensitive indicator *Escherichia coli *(7.5–15 mM). Lactobacilli and *Clostridium clostridioforme *were more resistant with MIC ranging from 15 to 50 mM. No correlation between bacterial intracellular concentrations of LMW-SH, including glutathione, and reuterin or hydrogen peroxide sensitivities were found.

**Conclusion:**

Our data showed that intestinal bacteria were very sensitive to reuterin and that their intracellular concentration of LMW-SH was not directly linked to their capacity to resist reuterin or hydrogen peroxide. This suggests that detoxification by LMW-SH such as glutathione is not a general mechanism and that other mechanisms are probably involved in bacterial tolerance to reuterin and hydrogene peroxide.

## Background

*Lactobacillus reuteri *is a heterofermentative lactic acid bacteria that belongs to the authochtonous microbiota of humans and animals [1]. Animal studies have shown that certain *Lb. reuteri *strains used as probiotics can provide protection against detrimental effects of certain microbiological, chemical and physical stressors; lower cholesterol; modulate immune responses; and improve the development of ileal tissue [2-4]. *Lb. reuteri *SD2112 (ATCC 55730) has been shown to reduce the incidence and severity of diarrhoea of different origins in children and adults [5-7], and to survive the passage through the stomach and upper intestine and transiently colonize the human gastrointestinal tract [6]. Indeed, this strain has been used for more than ten years as probiotic and/or starter culture in food and health care products [4].

In the presence of glycerol *Lb. reuteri *can synthesize 3-hydroxypropionaldehyde (3-HPA). 3-HPA is excreted into the medium where it forms together with the hydrate and the dimer (reuterin), a dynamic multi-component equilibrium (HPA, HPA system: named reuterin in most publications) [4]. Reuterin is a potent antimicrobial agent active against Gram positive and Gram negative bacteria, as well as yeasts, moulds and protozoa [8]. Reuterin is synthesized *in vitro *under pH, temperature and anaerobic conditions similar to those of the gastrointestinal tract [9]. *In vivo*, active reuterin synthesis could occur in the colon via *Lb. reuteri *metabolism, if sufficient amounts of glycerol become available as a product of luminal microbial fermentations, digestion of luminal fats, sloughed mucus and desquamated epithelial cells, and intestinal clearing of endogenous plasma glycerol [2]. To our knowledge, there are presently no data concerning the amount of glycerol available for bacterial conversion to reuterin in the human intestine. 

Reuterin is water soluble, effective in a wide range of pH, resistant to proteolytic and lipolytic enzymes and has been therefore studied as a food preservative or auxiliary therapeutic agent [4,8,10]. Reuterin is also believed to play a role in the probiotic effects of *Lb*. *reuteri *SD2112, but its mode of action on microbial growth has not been elucidated due to the high complexity of the HPA-system chemistry [4]. It has been postulated that reuterin might inhibit the activity of bacterial ribonucleotide reductase, an enzyme catalyzing the first step in DNA synthesis, by competition (HPA-dimer) with ribonucleotides for binding sites or by reaction (3-HPA) with unstable sulfhydryl groups of ribonucleotide reductase or with thioredoxin which is required for enzymatic activity [11]. The inhibition of the conversion of ribonucleotides to deoxyribonucleotides would explain the broad-spectrum activity of reuterin [11]. It has also been suggested that the mechanism of action of reuterin might be directed towards sulfhydryl enzymes, and that the tolerance of bacteria to reuterin might be associated with intracellular levels of LMW-SH such as glutathione (unpublishd data).

Reduced glutathione (GSH), is a cysteine-containing tripeptide, mostly found in Gram negative bacteria such as purple and cyanobacteria. The highest GSH concentrations are observed in aerobes or facultative anaerobes, whereas strict anaerobes are generally deprived of GSH but possess other soluble thiol compounds [12]. However, some faculative anaerobic Gram positive bacteria (eg. *Lactococcus lactis *subsp. *lactis*, *Streptococcus agalactiae *ATCC 12927 or *Enterococcus faecalis *ATCC 29212) can synthesize significant amount of GSH, whereas others (e.g. *Lactococcus lactis *subsp. *cremoris *Z8 or *Streptococcus mutans *ATCC 33402) can import GSH from the growth medium, but this faculty is strain dependent [13,14]. GSH plays important functions in bacteria including control of redox potential, protection against oxidative stress, detoxification of endogenously and exogenously derived toxins, protein folding and storage and transport of organic sulfur [15]. This potent antioxidant is also believed to play a key role in detoxification of reactive aldehydes, such as acrolein and protects cells from oxidative damage such as produced by H_2_O_2 _[16]. Recently, glutathione reductase has been shown to contribute to *Lactobacillus sanfranciscensis *oxygen tolerance [17]. Various studies have reported the MIC of reuterin against pathogens and lactic acid bacteria [9,11,18]. However, the activity of pure reuterin against intestinal bacteria has never been reported, even though this data is important for understanding the probiotic effects of *Lb. reuteri in vivo *when administered to humans and animals. The aim of this study was therefore to determine the *in vitro *antibacterial activity of pure reuterin on a representative panel of human intestinal bacteria. The intracellular concentration of LMW-SH and the sensitivity of intestinal bacteria to hydrogen peroxide were also recorded to tentatively correlate reuterin sensitivity to intracellular LMW-SH such as GSH and oxygen tolerance.

## Results

### Production and purification of reuterin

**Figure 1:**
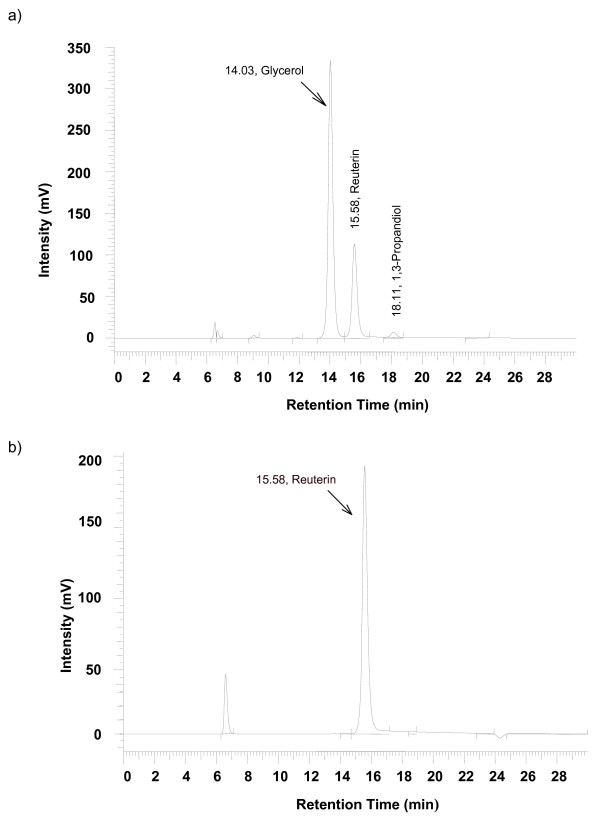
**HPLC chromatograms**. a) supernatant before purification; b) reuterin after purification on a silica gel 60 chromatography column with acetone:ethyl acetate (2:1) as eluent.

Approximately 170 mM reuterin was produced by *Lb. reuteri *SD2112 from 200 mM glycerol. After purification of 140 ml of this solution, about 1 g of pure reuterin was recovered, corresponding to a yield of 57 %. A high purity of reuterin, free from contaminants such as glycerol and 1,3 propanediol, was obtained after the purification process, as shown by HPLC analysis (Figure 1).

### Antimicrobial activity

The MIC and MBC of the tested bacteria are presented in Table 1. As expected, *Lb. reuteri *was the most resistant species, with MIC and MBC ranges for both tested strains of 30–50 mM and 60–120 mM, respectively, followed by other lactobacilli (MIC range 15–40 mM; MBC range 15–80 mM). The MIC and MBC for the sensitive strain *Escherichia coli *were in the range from 7 to 15 mM and from 15 to 30 mM, respectively.

**Table 1:** Reuterin activity and LMW-SH concentration in intestinal bacteria

**Organisms**	**Strains^a^**	**MIC^b^[mM]**	**MBC^b ^[mM]**	**LMW-SH^c ^[μmol/10^12 ^cells]**
***Escherichia coli***	DSM 5698	7.5–15.0	15.0–30.0	7.9 ± 1.3
***Bacteroides vulgatus***	DSM 1447	< 1.9	1.9–3.8	1.6 ± 0.4
***Bacteroides thetaiotaomicron***	DSM 2079	1.9–3.8	1.9–3.8	-^d^
***Bacteroides fragilis***	LMG 10263	3.8–7.5	3.8–7.5	7.3 ± 1.7
***Bifidobacterium catenulatum***	LMG 11043	1.9–3.8	7.5–15.0	1.8 ± 0.9
***Bifidobacterium longum***	DSM 20219	1.9–3.8	3.8–7.5	1.5 ± 0.5
***Bifidobacterium longum infantis***	DSM 20088	1.9–3.8	1.9–3.8	5.2 ± 0.6
***Bifidobacterium adolescentis***	DSM 20083	3.8–7.5	-^d^	1.3 ± 0.1
***Bifidobacterium bifidum***	DSM 20456	3.8–7.5	7.5–15.0	2.4 ± 0.5
***Bifidobacterium breve***	DSM 20213	7.5–15	7.5–15.0	2.0 ± 0.1
***Lactobacillus acidophilus***	ATCC 4356	15.0–40.0	15.0–40.0	10.7 ± 0.3
***Lactobacillus casei***	ATCC 334	15.0–40.0	40.0–80.0	2.0 ± 0.3
***Lactobacillus fermentum***	ETH-Z	15.0–40.0	15.0–40.0	2.9 ± 1.2
***Lactobacillus salivarius***	ETH-Z	15.0–40.0	40.0–80.0	22.2 ± 5.0
***Lactobacillus reuteri***	DSM 20016	30.0–50.0	60–120.	6.5 ± 0.8
***Lactobacillus reuteri***	SD 2112	30.0–50.0	60–120	6.1 ± 1.4
***Eubacterium biforme***	DSM 3989	1.9–3.8	3.8–7.5	3.0 ± 0.7
***Eubacterium eligens***	DSM 3376	1.9–3.8	1.9–3.8	-^d^
***Colinsella aerofaciens***	DSM 3979	3.8–7.5	7.5–15.0	21.9 ± 4.3
***Enterococcus faecium***	DSM 20477	3.8–7.5	30.0–50.0	4.8 ± 1.3
***Streptococcus salivarius***	DSM 20560	3.8–7.5	15.0–30.0	9.6 ± 1.1
***Clostridium difficile***	ETH-Z	< 1.9	3.8–7.5	43.0 ± 3.8
***Clostridium clostridioforme***	DSM 933	15.0–30.0	15.0–30.0	3.1 ± 0.8
***Ruminococcus productus***	DSM 2950	7.5–15.0	3.8–7.5	2.5 ± 0.5
***Listeria innocua***	HPB 13	7.5–15.0	7.5–15.0	5.5 ± 1.4
***Listeria ivanovii***	HPB 28	3.8–7.5	7.5–15.0	1.5 ± 0.6

^a^DSM: Deutsche Sammlung von Mikroorganismen und Zellkulturen, Braunschweig, Germany ATCC: American Type Culture Collection, Rockville, Maryland, USA HPB: Health Protection Branch, Health and Welfare Canada, Ottawa, Ontario, Canada ETH-Z:Culture Collection of the Food Biotechnology Laboratory, ETH Zürich, Switzerland LMG: Belgian Co-Ordinated Collections of Micro-Organisms, Gent, Belgium SD2112 = BioGaia, Stockholm, Sweden

^b^MIC and MBC data are expressed as the range obtained from at least three independent repetitions for each species

^c^LMW-SH concentrations are expressed as mean ± SD of at least three independent repetitions

^d^Not determined

Among the 19 (non lactobacilli) tested species, 15 showed MIC values below that measured for the indicator *E. coli *strain with MIC equal or below 7.5 mM. The most sensitive strains to reuterin were *Bacteroides vulgatus *and *Clostridium difficile *(MIC < 1.9 mM), followed by *Bacteroides thetaiotaomicron, Eubacterium eligens, Bifidobacterium longum *var *infantis, Eubacterium biforme, Bifidobacterium longum *and *Bifidobacterium catenulatum *(MIC range from 1.9 to 3.8 mM). The four most resistant non lactobacilli species were *Clostridium clostridioforme *(MIC range 15–30 mM; MBC 15–30 mM) followed by *Ruminococcus productus*, *Bifidobacterium breve *and *Listeria innocua *(MIC range 7.5–15 mM and MBC range 3.8–15 mM).

### Intracellular LMW-SH concentration including GSH

Intracellular concentrations of LMW-SH in tested bacteria are reported in Table 1. LMW-SH were detected in all bacteria and was in the range from 1.3 to 3.1 μmol/10^12 ^colony forming unit [cfu]) for many bacteria. The highest concentrations were found in *Cl. difficile *(43 μmol/10^12^cfu) followed by *Collinsella aerofaciens*, *Lactobacillus salivarius, Lactobacillus acidophilus *and *Streptococcus salivarius *(ranging from 22 to 10 μmol/10^12 ^cfu). *E. coli, Bacteroides fragilis*, *Lb. reuteri *(DSM 20016 and SD 2112),*L. innocua*, *B. longum *var *infantis *and *Enterococcus faecium *showed intermediate values ranging from 8 to 5 μmol/10^12 ^cfu).

### Sensitivity to H_2_O_2_

The sensitivity of several intestinal bacteria to H_2_O_2 _is shown in Table 2. For all tested bacteria, except for *E. faecium*, exposure to 10, 20 and 30 g l^-1 ^H_2_O_2 _caused a decrease in viable cell counts compared to the control medium without H_2_O_2_. Among the 25 tested, the 4 most resistant species were *E. faecium, Lb. acidophilus, Lb. reuteri *(DSM 20016) and *S. salivarius *with survival rates to 30 g l^-1 ^H_2_O_2 _ranging from 75.6 to 0.06%. The second group, *B. longum *var *infantis *and *Lactobacillus fermentum *survived to 20 g l^-1 ^H_2_O_2_(at 0.18 and 0.02 % respectively), whereas a third group with four bifidobacteria and three lactobacilli species showed survival rates to 10 g l^-1 ^H_2_O_2 _ranging from 0.07 to 24.9 %. The most sensitive group with complete growth inhibition with 10 g l^-1 ^of H_2_O_2_included 9 species belonging to known strict anaerobe genera from the human gut (*Bacteroides *spp., *Clostridium *spp., *C. aerofaciens, E. biforme, R. productus *and one bifidobacteria among 6 tested). Surprisingly, *Listeria *spp. and *E. coli*, classified as facultative anaerobes, were also completely inhibited with 10 g l^-1 ^H_2_O_2_.

**Table 2:** Bacterial survival rates (%) after exposure to different hydrogen peroxide concentrations

**Organisms**	**Strains**	**0 g l^-1^** **H_2_O_2_**	**10 gl^-1^** **H_2_O_2_**	**20 gl^-1^** **H_2_O_2_**	**30 gl^-1^** **H_2_O_2_**
***Escherichia coli***	DSM 5698	100	ng^1^	ng	ng
***Bacteroides vulgatus***	DSM 1447	100	ng	ng	ng
***Bacteroides thetaiotaomicron***	DSM 2079	100	ng	ng	ng
***Bacteroides fragilis***	LMG 10263	100	ng	ng	ng
***Bifidobacterium catenulatum***	LMG 11043	100	0.56^a^	ng	ng
***Bifidobacterium longum***	DSM 20219	100	0.47^a^	ng	ng
***Bifidobacterium longum infantis***	DSM 20088	100	5.90^b^	0.18^abc^	ng
***Bifidobacterium adolescentis***	DSM 20083	100	0.67^a^	ng	ng
***Bifidobacterium bifidum***	DSM 20456	100	ng	ng	ng
***Bifidobacterium breve***	DSM 20213	100	24.9^bc^	ng	ng
***Lactobacillus acidophilus***	ATCC 4356	100	25.5^bc^	3.83^bd^	3.88^a^
***Lactobacillus casei***	ATCC 334	100	0.07^a^	ng	ng
***Lactobacillus fermentum***	ETH-Z	100	0.72^a^	0.02^c^	ng
***Lactobacillus salivarius***	ETH-Z	100	0.71^a^	ng	ng
***Lactobacillus reuteri***	DSM 20016	100	0.35^a^	0.06^c^	0.09^b^
***Lactobacillus reuteri***	SD 2112	100	0.16^a^	ng	ng
***Eubacterium biforme***	DSM 3989	100	ng	ng	ng
***Colinsella aerofaciens***	DSM 3979	100	ng	ng	ng
***Enterococcus faecium***	DSM 20477	100	66.1^c^	48.4^d^	75.6^c^
***Streptococcus salivarius***	DSM 20560	100	0.57^a^	0.20^abc^	0.06^b^
***Clostridium difficile***	ETH-Z	100	ng	ng	ng
***Clostridium clostridioforme***	DSM 933	100	ng	ng	ng
***Ruminococcus productus***	DSM 2950	100	ng	ng	ng
***Listeria innocua***	HPB 13	100	ng	ng	ng
***Listeria ivanovii***	HPB 28	100	ng	ng	ng

^1^No growth

Different letters in a line indicate significant differences with the Tukey's test (*P *< 0.05)

## Discussion

In this study, the MIC and MBC of pure reuterin for different intestinal bacteria were determined using a serial dilution microtiter plate assay. With the exception of Dobrogosz *et al*. [19], who express the inhibitory activity of reuterin with arbitrary units defined, after analysis with ^14^C-labeled glycerol, as 1 U equal 8 μg reuterin/ml, most of the authors have used poorly defined arbitrary units to express the inhibitory concentration of reuterin [10,20]. Therefore, a direct comparison of published data for antimicrobial activities of reuterin against different bacteria is difficult. Another problem of these studies is that the molar concentration of reuterin was calculated using the molecular mass of the dimer form [9,11,21,22], although reuterin occurs in its monomeric form at the low concentrations used [4]. Moreover arbitrary units are changed by many experimental factors as shown for bacteriocins, such as variation of sensitivity among indicator strains of the same species, medium and incubation conditions for the activity tests and concentrations of antimicrobial compounds in tested preparations [23,24]. For data comparison, test conditions must be standardized and MIC data expressed in μg ml^-1 ^or mM. In this study the molar concentration of reuterin was determined by a method originally developed for acrolein quantification, using the acid-catalyzed formation of a colored complex between acrolein/HPA and tryptophan [25], which has been adopted by several other authors to correctly quantify reuterin [4,26-28]. Furthermore, all bacteria were tested under controlled test conditions and the same medium for the activity test.

Our data showed that reuterin is an effective inhibitor for most tested intestinal bacteria, with the exception of lactobacilli. Among the 20 species non belonging to the *Lactobacillus *genus, 14 were inhibited and/or killed by reuterin concentrations equal or below 3.8 mM. They exhibited higher sensitivity than *E. coli*, the usual sensitive indicator used to test reuterin [4]. Dobrogosz *et al*. [11] and Chung *et al*. [9] reported, using a two-fold dilution test, MIC for *E. coli *K12 of 4–5 U/ml (0.9–1.1 mM, after recalculation based on 3-HPA monomer and 1 U = 8 μg reuterin/ml), 32 U/ml (6.9 mM) for *Lb. reuteri *and ranging from 4 to 42 U/ml (0.9–9.1 mM) for different lactobacilli. Our data show the same trends; MIC values for lactobacilli were at least 2 times higher than for *E. coli *(Table 1). However, under our test conditions, MIC measured for lactobacilli were approximately 10 times higher than reported in previous studies. Dobrogosz *et al*. [11] found that *Listeria monocytogenes *showed a similar low sensitivity to reuterin as for lactobacilli, with MIC of 4–42 U/ml (0.9–9.1 mM), which was different from our data for *L. ivanovii *and *L. innocua*, which were much more sensitive to reuterin than lactobacilli. These differences for MIC measured in different studies can be due to various factors of the arbitrary activity tests, including pH, culture media, incubation temperature, concentration and physiological state of the tested bacteria which affect both the growth [23] of the tested strains and the activity of reuterin [27]. As well, a strict criteria for MIC calculation (lowest reuterin concentration resulting in complete inhibition of the tested strain) was used in our study. The aldehyde function of 3-HPA is highly reactive due to its β-hydroxy moiety and therefore the molecule spontaneously reacts with available amino- and sulfhydryl-functional groups present in compounds of the growth medium and bacteria [4]. According to Lüthi-Peng *et al*. [27], only 30 % of reuterin remains free after 24 h of incubation at 37°C in a complex medium. The lactobacilli were also tested in the LAMVAB growth medium [29], which is used as selective medium to enumerate lactobacilli in fecal samples, at pH 5.0. With this medium MIC were 2–5 times lower than the MIC obtained with supplemented BHI (data not shown). This result clearly shows that the comparison of data from different studies on reuterin activity is difficult, but comparison within one study is much more valid. This limitation should also be taken into account when sensitivity data to antimicrobial compounds such as reuterin obtained with *in vitro *tests, are extrapolated to other *in vitro *systems or *in vivo *conditions such as in the gastrointestinal tract.

To tentatively explain the different behavior of intestinal bacteria toward reuterin activity, we analyzed their intracellular concentration of LMW-SH including GSH, which has been shown to be involved in aldehyde detoxification mechanisms [30]. With this intention, we used the Ellmann's reagent that specifically reacts with thiol groups. To make the distinction between LMW-SH, and thiol groups of proteins, the concentration of intracellular LMW-SH was determined in the cell-free supernatant of bacterial crude extracts after protein elimination by precipitation with sulfosalicylcylic acid. Although, all LMW-SH and not only GSH were measured in the present study, this test gives a good estimation of GSH concentration because other LMW-SH occur only in low concentration [31]. LMW-SH were present in all tested bacteria. The highest intracellular concentrations (> 20 μmol/10^12 ^cells) were measured in two Gram positive strict anaerobic species (*C. aerofaciens *and *Cl. difficile*) and one facultative anaerobic bacteria (*Lb. salivarius*). Although glutathione appears to be mostly present in cyanobacteria and purple bacteria [12], high intracellular glutathione concentrations have already been measured in Gram positive Closdriales, including strict anaerobes such as *Clostridium perfringens *[32] and *Lactobacillus *spp. [33,34] and *Bifidobacterium *spp. [35]. It is usually suggested that Gram positive organisms do not produce GSH but accumulate it from the growth medium. However, other evidences indicate that some Gram positive bacteria (e.g. *Enterococcus faecalis *ATCC 29212 or *Streptococcus agalactiae *ATCC 12927) can synthetize GSH [13]. Moreover, Fernandes and Steele [33] showed that intracellular GSH levels in lactic acid bacteria varied widely between organisms and were very dependent on strains tested as well as growth conditions, including growth medium composition (presence or absence of GSH or precusors) and aeration, as well as bacterial growth phase. Very few data are reported on the capacity of intestinal bacteria to synthesize or harvest GSH from the growth medium. It has been shown that some probiotic lactobacilli strains can produce and even release high amount of GSH [36]. In our study, the growth medium was deprived of cystein, which is a major precursor for GSH synthesis. However, GSH from yeast extract was likely available for bacterial uptake in the growth medium [12]. We recently observed that external addition of GSH could conteract the toxicity of reuterin (3-HPA) toward *E. coli *K12, which suggests a correlation between induced toxicity of 3-HPA on *E. coli *K12 and depletion of GSH (unpublished data). However, in this study, the lack of correlation between intracellular GSH concentration and reuterin sensitivity suggests that other detoxification mechanisms prevent 3-HPA toxicity, such as 1,3-propanediol-dehydrogenase, which converts 3-HPA to the unreactive 1,3-propanediol. No inhibitory activity of 1,3-propanediol against intestinal bacteria is known until now.

GSH is considered to play a key role in protecting cells against oxidative stress. It is usually the most abundant intracellular, low-molecular-weight thiol in *E. coli *[32] and might contribute to protect *E. coli *cells [30] as well as Gram positive bacteria [34] from the lethal effects of oxidative damage. It is known that GSH can reduce hydrogen peroxide in exchange for oxidation of GSH to glutathione disulfide (GSSG) [30]. The tolerance of intestinal bacteria to hydrogen peroxide was then determined to assess a possible correlation between their sensitivity to reuterin and oxygen detoxification. Oxygen toxicity is considered to result from the effects of activated compounds including superoxide, hydrogen peroxide and hydroxyl radicals [37]. Facultative anaerobic bacteria decompose and detoxify these activated oxygen metabolites enzymatically with superoxide dismutase and catalase [37]. Most of the intestinal bacteria, classified as strict anaerobic organisms, are devoid of these enzymes and therefore their growth can be inhibited when H_2_O_2 _is present [37]. Indeed, the most oxygen sensitive species tested, which belongs to the orders Bacteroidales (*Bacteroides *spp.) and Clostridiales (*Clostridium *spp., *Ruminococcus *sp., *Eubacterium *spp. and *Collinsella *sp.) were completely inhibited with 10 g l^-1 ^hydrogen peroxide. Five bifidobacteria among the six tested, were not inhibited by 10 g l^-1 ^hydrogen peroxide although they are classified as a strict anaerobic bacteria. Using the same technique, Shimamura *et al*. [37] reported variations in H_2_O_2 _sensitivity of *Bifidobacterium *spp., and found similar results with *B. infantis *being the least sensitive to H_2_O_2_, followed by *B. breve, B. adolescentis *and *B. longum*. The facultative anaerobic bacteria tested were found to largely differ in their sensitivities to hydrogen peroxide. Among lactobacilli, *Lb. acidophilus *and *Lb. reuteri *DSM 20016 were the most tolerant.*Lb. acidophilus *and *Bifidobacterium *spp. have NADH oxidase and NADH peroxidase to scavenge hydrogen peroxide and prevent cell death in the presence of oxygen [37,38]. Moreover, it is known that some *Lb. acidophilus *strains produce hydrogen peroxide and peroxidase [39]. In addition, certain lactobacilli produce superoxide dismutase [40] and a non-heme pseudocatalase [41].

Surprinsigly, *E. coli *and *Listeria *spp. also classified as facultative anaerobes and which possess a catalase were inhibited by 10 g l^-1 ^hydrogen peroxide. But it has also been shown that the high H_2_O_2 _sensitivity of *E. coli *can be related to DNA damage caused by hydrogen peroxide [42]. In this study, we did not establish any correlation between bacterial hydrogen peroxide sensitivity and intracellular LMW-SH concentration or reuterin sensitivity. However, the wide range of organisms tested an the multiplicity of oxygen detoxification mechanisms as well as the wide functions of LMW-SH and GSH in microorganisms [15] suggest that strain specific mechanisms are likely to be involved rather than a general mechanism applicable to all bacteria.

## Conclusion

This study reports for the first time the antimicrobial activity of reuterin on various intestinal bacteria. Large differences among species were observed. With the exception of *Lactobacillus *strains which were more resistant, most intestinal bacteria were inhibited by reuterin concentration below 7.5 mM. Moreover, the MBC (7.5–15 mM) recorded for the two *Listeria *spp. tested were higher than for the main intestinal commensals. The most sensitive species were inhibited by reuterin concentration around 2 mM, which suggests that low concentration of reuterin production *in situ *(if sufficient amount are present either via *in situ *production or ingested as encapulated form with a probiotioc bacteria) seems to be sufficient to alter the intestinal bacterial growth. However caution should be applied when extrapolating these results obtained with *in vitro *activity tests to specific *in vivo *conditions, where other important factors such as nutrient competition and antagonistic and/or synergistic relationships between the intestinal microbiota can largely influence the inhibition effects of antimicrobials such as reuterin. Complementary experiments are therefore needed in more complex *in vitro *systems, such as *in vitro *intestinal fermentation models, as *in vivo *experimentation is very difficult. No correlation was found between bacterial sensivity to reuterin and intracellular LMW-SH and/or hydrogen peroxide sensitivity. Our data suggest that protection via LMW-SH such as GSH is not a general protection mechanism toward reuterin toxicity.

## Methods

### Chemicals

If not otherwise stated, all chemicals were obtained from Fluka, Buchs, Switzerland.

### Bacterial strains, media and growth conditions

The bacteria used in this study are shown in Table 1. All strains were kept frozen at -80°C in 25 % (v/v) glycerol supplemented with L-cysteine (0.05 %). Before use, intestinal bacteria were sub-cultured at least twice at 24 h intervals in brain heart infusion (BHI, Biolife, Milano, Italy) medium supplemented with yeast extract (5 g/l), K_2_HPO_4 _(1 g/l), glucose (8 g/l), Tween 80 (1 ml/l), hemin (0.005 g/l), vitamin K1 (0.002 g/l) and titanium citrate (0.6 mM) as reduction agent, at 37°C (pH 6.6) under N_2_and CO_2 _gas atmosphere (1.5 bar, 80:20 [v/v]). Tested bacteria were incubated between 14 and 18 h at 37°C. *Lb. reuteri *SD2112 (ATCC 55730) that was used to produce reuterin [27] was grown at 37°C in MRS broth [43] from Difco Laboratories (Milan, Italy).

### Reuterin production

Reuterin was produced as previously described by Vollenweider *et al*. [44]. Briefly, *Lb. reuteri *was inoculated at 1% (v/v) in 10 ml MRS broth, incubated overnight at 37°C and added to 50 ml MRS medium which was then incubated for 3 h at 37°C. This culture was added to 1 L of MRS medium supplemented with 20 mM glycerol and incubated overnight. The cells were then harvested by centrifugation at 1500 × *g *for 10 min at 20°C, washed with potassium phosphate buffer (0.1 M, pH 7.0), resuspended in 300-ml sterile aqueous solution of glycerol (200 mM) and incubated for 2 h at 37°C. The cells were removed by centrifugation (8000 × *g*, 10 min), and 140 ml of the supernatant was filter-sterilized (FP/30/0.2 CA-S; Schleicher & Schuell GmbH, Einbeck, Germany) and lyophilized. Reuterin was purified on a silica gel 60 chromatography column (Merck, Darmstadt, Germany), with acetone:ethyl acetate (2:1) as eluent. The 3-HPA solution was diluted with distilled water to obtain a 10 M solution, which was stable for at least 6 months at 4°C. The concentration and purity of 3-HPA was verified by colorimetric method and HPLC analysis as described below.

### 3-HPA quantification

The concentration of reuterin in the tested solution was determined with the assay for 3-HPA content, based on the colorimetric method of Circle *et al*. [25], using acrolein for calibration as described by Vollenweider *et al*. [44]. The solution of 3-HPA was also analyzed by HPLC (Hitachi LaChrome, Merck, Dietikon, Switzerland) on an Aminex HPX-87H column (300 × 7.8 mm, Bio Rad, Reinach, Switzerland) with 10 mM H_2_SO_4 _as eluent and a flow rate of 0.6 ml min^-1 ^[45].

### Antimicrobial activity

The minimal inhibitory (MIC) and bactericidal (MBC) concentrations of 3-HPA were determined using a microtiter assay based on the method described by Mota-Meira *et al*. [46] with modification. All manipulations were done in an anaerobic chamber under an atmosphere of nitrogen containing 5 % (v/v) hydrogen. Briefly, bacteria were grown anaerobically in supplemented BHI at 37°C for 15 to 18 h. The optical density (OD_590 nm_) was adjusted to 0.1 with fresh supplemented BHI medium using a spectrophotometer (1420 Multilabel Counter, Wallac, PerkinElmer, Schwerzenbach, Switzerland). This OD_590 nm _corresponds approximately to a 0.5 McFarland standard (National Committee for Clinical Laboratory Standards, 1991). Before each test, a working solution of reuterin (300 mM, in supplemented BHI) was prepared from the stock solution (10 M). One hundred microliter of the working reuterin solution was added in the first row of a 96-well microtiter plates (tissue culture plate, flat bottom, Greiner Bio-one, St-Gallen, Switzerland) containing 100 μl of supplemented BHI broth and a serial two-fold dilution was done. Reuterin was not added to the last well of a column which was used as positive growth control. Each well was then inoculated with 25 μl of the OD-standardized bacterial suspension. The microtiter plates were incubated in anaerobic jars with an atmosphere generation system (Oxoid AnaeroGenTM, Oxoid, Bâle, Switzerland) at 37°C under static conditions until the positive growth control (tested bacteria grown without reuterin) showed clear bacterial growth (OD_590 nm _≥ 0.8). After incubation, the OD_590 nm _was measured using a microtiterplate reader. Non-inoculated supplemented BHI broth with added inhibitor, incubated under the same conditions, was used as a blank. *E. coli *K12 was used as indicator organism due to its high sensitivity to reuterin [11]. The MIC was calculated from the highest dilution showing a complete inhibition of the tested strain (OD_590 nm _equal to the OD_590 nm _of the blank). The MBC, corresponding to the concentration that killed 99.9 % of the initial inoculum [47], was determined by spotting 20 μl from a dilution row done with the samples from first wells showing no visible growth on supplemented BHI-agar. The MBC was defined as the lowest concentration at which no growth on the agar plate was observed [47]. Agar plates were incubated anaerobically for 48 h in jars. The MBC of reuterin was expressed in mM. Each organism was tested between 3 to 15 times independently, and the results are expressed as ranges of MIC or MBC expressed in mM.

### Determination of LMW-SH including GSH

The intracellular content of LMW-SH-groups was determined in deproteinized cell-free extracts of bacterial cultures by the colorimetric method of Ellmann [48], with some modifications. Bacteria were inoculated at 1% (v/v) in supplemented BHI and incubated overnight in sealed bottles under gas atmosphere of N_2 _and CO_2 _(1.5 bar, 80:20 [v/v]) at 37°C. Bacterial cell counts were determined by plating on supplemented BHI agar. Fifteen ml of the bacterial cultures were centrifuged at 3000 × *g *for 10 min and pellets were carefully resuspended in 300 μl of B-PER™ solution. A volume of 130 μl of this solution was mixed with 130 μl of 5-SSA (2.5 % w/v). The mixture was incubated for 15 min at 4°C and the precipitated proteins were removed by centrifugation (10000 × *g *for 15 min at 4°C). A 150 μl volume of the resulting supernatant was mixed with 840 μl of potassium buffer (0.2 M, pH 7.5) and 10 μl of Ellmann's reagent, and incubated 10 min at room temperature. The absorption was measured at 412 nm (Uvikon 922, Kontron Instrument, Milan, Italy). The concentration was expressed as μmoles LMW-SH-groups per 10^12 ^total bacterial cells. A standard curve was done with various concentrations (0, 20, 40, 60, 80 μM) of glutathione in potassium phosphate buffer (0.2 M, pH 7.5) that were added to a 75 μl/ml B-PER™ solution (Bacterial Protein Extraction Reagent, Pierce Chemical Company, Rockford, Illinois, USA) and the same amount of 5-sulfosalicylcylic acid-dihydrate (2.5 % w/v, 5-SSA) for GSH determination, giving a final volume of 1 ml. After mixing carefully, 10 μl of Ellmann's reagent (100.9 mM 5,5'-dithio-bis(2-nitrobenzoic acid) (DTNB) in dimethyl sulfoxide) was added and the mixture was incubated 10 min at room temperature. The absorption was measured at 412 nm. Reported data are means of triplicate analyses.

### Tolerance to hydrogen peroxide

Cell survival to hydrogen peroxide was determined as previously described by Doleyres *et al*. [49] with some modifications. Briefly, 100 μl of an overnight culture of the tested bacteria was added to 900 μl of H_2_O_2 _solution at different final concentrations (0, 10, 20 or 30 g l^-1^), and incubated for 1 min at 37°C. After incubation, 10 μl of tested sample was added to 490 μl of a catalase solution (168640 U/ml) to stop the reaction. Cell survival was determined by plating the appropriate dilution on supplemented BHI-agar and incubating 48 h at 37°C. All the manipulations were done in an anaerobic chamber (Coy Laboratories, Ann Arbor, MI, USA) under an atmosphere of nitrogen containing 5 % (v/v) hydrogen. Reported data and standard deviation are calculated from triplicate analyses.

### Statistics

Data for the survival test to hydrogen peroxide did not meet assumptions of ANOVA (normally distributed and independent residuals) and were therefore transformed before analysis.

Values for survival rates after treatments with 10 and 30 g l^-1^H_2_O_2 _were transformed by log_10 _function after adding 1. Values for survival rates after treatment with 20 g l^-1 ^H_2_O_2 _were divided by 10 and transformed by log_10 _function. Treatment means were compared using the Tukey's test with the probability level of *P *< 0.05. The Pearson's correlation was calculated between reuterin activity, tolerance to H_2_O_2_, and LMW-SH concentrations in bacterial cells using SPSS 13.0 for Windows (SPSS Inc., Chicago, IL, USA).

## Authors' contributions

GLB, CL and VC designed the experiments. SV performed the production and purification of reuterin. VC and MD performed the activity test experiments. VC performed the determination of LMW-SH and the cell survival to hydrogen peroxide. GLB, CL and SV revised the manuscript. All authors read and approved the final manuscript.
